# Three-dimensional assessment of low-level laser therapy on orthodontic miniscrew displacement using CBCT: a retrospective study

**DOI:** 10.1186/s12903-024-04711-x

**Published:** 2024-08-24

**Authors:** Dina Alaaeldin Elfouly, Sherief Hussein Abdel-Haffiez, Nadia Mosaad El-Harouni, Mohamed Abdel Sattar Elzoheiry, Eiman Salah Marzouk

**Affiliations:** Department of Orthodontics, Faculty of Dentistry, Alexandria University, Champollion St., P.O. Box 21521, Azarita, Alexandria, Egypt

**Keywords:** Miniscrews, Displacement, Low level laser, 3D, CBCT

## Abstract

**Background:**

This study aimed to assess the effect of Low-Level Laser Therapy (LLLT) on sagittal, transverse and vertical Orthodontic miniscrew displacement.

**Materials and methods:**

The study included CBCTs from the records of 12 adult patients who underwent upper first premolar extraction and canine retraction with orthodontic miniscrews for maximum anchorage. The miniscrews on one side received LLL, while the other side served as a control. The Low-Level Laser was applied to assess its effect on the displacement of the miniscrews. The used CBCTs have been taken at two-time points: immediately after miniscrew insertion (T0) and four months after the start of canine retraction (T1) with a total of 24 CBCTs. Miniscrew displacement was assessed by measuring head (HMS) and tail (TMS) displacement to the axial, coronal and mid-sagittal planes on the CBCT at the two time points. Miniscrews displacement (T1-T0) was compared between LLL side and control side. Comparisons were performed using paired samples t-test. The significance level was set at *p*-value < 0.05. The reproducibility of measurements was assessed by intraclass correlation coefficient (ICC).

**Results:**

After four months of canine retraction, HMS and TMS from both laser and control sides showed significant three-dimensional displacement at *p* < 0.05. No significant difference in mean displacement in the vertical, sagittal, nor transverse planes between both sides was elicited.

**Conclusion:**

LLL application in the used protocol does not affect the amount of miniscrew displacement in any of the three planes of space. Miniscrew displacement was significant in both groups.

## Introduction

The fundamental necessity for correcting various forms of malocclusions is secured anchorage. For many years, anchorage needs were met by teeth and intramaxillary appliances [1].

The introduction of temporary Anchorage Devices (TADs) in orthodontics has been particularly significant in this regard, as they provide reliable and predictable anchorage, thereby enhancing the overall effectiveness and efficiency of orthodontic treatments [2]. TADs have gained increasing popularity both in clinical practice and academic research over the past two decades [3].

TADs are affordable, easily inserted and removed, and available in a variety of beneficial sizes [4]. Miniscrews can provide a secure anchorage for various tooth movements, including retraction, intrusion, protraction [5], extrusion [6] and distalization [7]. Nevertheless, obtaining primary stability poses a challenge in their clinical application [8].

Primary stability of miniscrew is the mechanical contact between the surface of miniscrew and bone and it is very crucial as the lack of primary stability leads to mobility of the miniscrew and eventually its loss [9]. Some authors reported that miniscrews move when submitted to orthodontic force [10, 11]. This movement because of remodelling processes is known as secondary displacement. This displacement can cause miniscrews contact vital oral structures, including root surfaces or even blood vessels and nerves [10].

Low Level Laser Therapy (LLLT) has various uses in dentistry including; accelerating tooth movement [12], reducing pain [13], and improving alveolar bone repair by reducing inflammation [14]. Moreover, LLLT is suggested to prevent miniscrew displacement. Previous research revealed that LLLT had a stabilising effect on miniscrews following direct application as measured by resonance frequency analysis or periotest [12, 15]. Cone beam computed tomography (CBCT) is a commonly used diagnostic tool for assessing the displacement of miniscrews following orthodontic loading [16, 17].

In contrast to traditional radiography techniques, which use X-rays to project structures onto a one-dimensional (1D) plane, CBCT provides cross-sectional pictures, and allows for the investigation of structural relationships using 2D scrolling or 3D volume rendering [18]. Additionally, it permits the visualization of structures without superposition or magnification, providing measurements with high reliability and accuracy [19].

To our knowledge, no previous studies assessed the displacement of miniscrews after LLL application three dimensionally, maybe due to the belief that the miniscrew will only migrate mesially if it moves. Thus, the objective of this study was to assess the sagittal, transverse, and vertical miniscrew movement after LLL application using CBCT.

Null Hypothesis: There is no difference between the LLL-treated group and the non-treated group in the displacement of miniscrews.

## Materials and methods

### Design and registration

A retrospective design was conducted after the approval of the Institutional Review Board at the Alexandria Faculty of Dentistry, Alexandria, Egypt (IRB:00010556–IORG:0008839). Manuscript Ethics Committee number (0881-02/2024). Orthodontic records of patients treated at our institute were used in the study according to the recommended sample size. All the research procedures were performed in accordance with the relevant guidelines and regulations, as stated in the Declaration of Helsinki.

### Sample size and statistical analysis

The 95% confidence level was used to determine the sample size needed to detect differences in orthodontic miniscrew displacement between the LLLT and control sides. Osman et al. [12] reported estimates of miniscrew displacement. The minimum sample size was calculated to be 11 patients (22 sides) increased to 12 (24 sides) to make up for procedural problems [20]. The sample size estimation was carried out using MedCalc Statistical Software version 19.0.5 (MedCalc Software bvba, Ostend, Belgium; https://www.medcalc.org; 2019).

The sample consisted of CBCT records of 12 patients taken immediately after miniscrews insertion (T0) and four months after starting canine retraction (T1). These records were of patients treated by the same health professional (M.Z) using the same treatment protocol.

### Participants and study settings

Inclusion criteria for records selection included: (1) Permanent dentition. (2) Class I bimaxillary protrusion and Class II division1 patients who required upper first premolars extraction and canine retraction using orthodontic miniscrews for maximum anchorage. (3) CBCTs obtained immediately after miniscrew insertion (T0) and after four months of canine retraction (T1). While exclusion criteria included: (1) Previous orthodontic therapy. (2) Cleft lip and palate patients (3) Medically compromised patients. (4) Patients with conditions that might impair bone physiology, including chronic renal failure, and hormonal disorders as thyroid and parathyroid [21]. (5) Patients receiving drugs that affect bone physiology as steroids, barbiturates, anticonvulsants drugs, and thyroid hormone replacements [22]. (6) Tobacco smoking patients [23].

### Intervention

Patients (aged between 18 and 22 years) received right and left self-drilling miniscrews to maximize anchorage during canine retraction. Miniscrews with a diameter of 1.6 mm and a length of 8 mm (Hubit Co Ltd, Ojeon-Dong, Korea) were implanted 6–8 mm from the alveolar crest on the buccal side between the maxillary second premolar and first permanent molars [24]. One side received LLL application (910-nm at 1.7 watts for 60 s) using diode laser (Doctor smile, Lambda SpA- Italy) on miniscrew’s head covering the surrounding tissue in a non-contact manner. The experimental side received one laser application every 72 h, for a total of four laser applications during canine retraction while the contralateral side served as the control side, with no LLL application in a split mouth design. Canine retraction was started two weeks after miniscrews insertion using closed coil spring (Modern orthodontics LLC, Morgan Hill, USA) delivering150gm of force on both sides.

All CBCT images were taken using the same CBCT machine (I-CAT Next generation device (Imaging Sciences International, Hatfield, Pa, USA)) at Faculty of Dentistry, Alexandria University. All scans were performed with the following parameters: 90 Kvp, 8 mA, W 100 mm x H 50 mm FOV (field of view) and 20 s scanning time at 0.25 resolutions, with the subjects’ heads positioned such that the Frankfort horizontal plane parallel to the floor. Images were stored as Digital Imaging and Communications in Medicine (DICOM) format.

### CBCT analysis

**Fig. 1:**
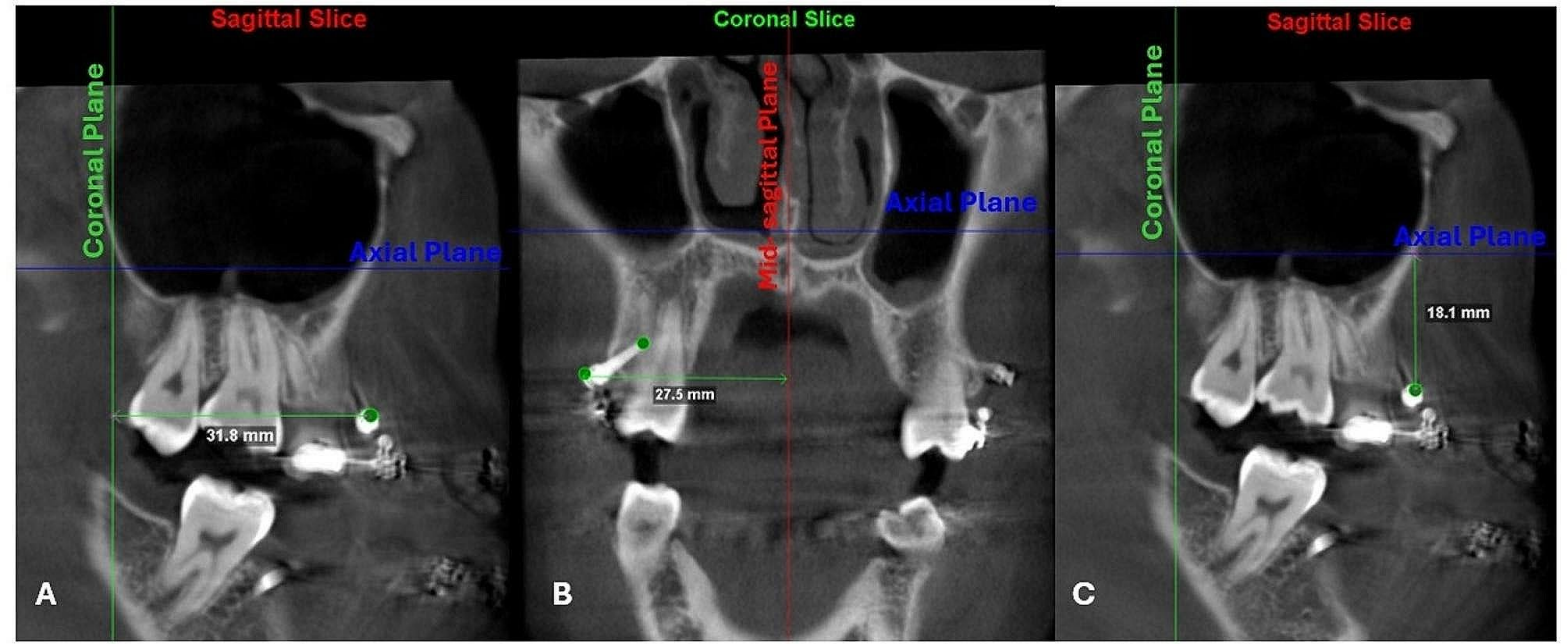
Cone beam computed tomography cross-sections showing the orientation of the three planes and measurement of the distances of minicrew’s head to planes: **A**, HMIC – coronal plane (green); **B**: HMIS – mid-sagittal plane (red); **C**, HMIA – axial plane (blue); (Dolphin Imagings software, version 11.95)

Assessment of the change in miniscrew’s head and tail position three dimensionally between the two time points T0 and T1. CBCTs at T0 (baseline for miniscrew initial position) and T1 (after four months of canine retraction) were analysed using Dolphin Imaging software v.11.95 Premium (Dolphin Imaging, Chatsworth, CA). First, each CBCT image was oriented in the sagittal view with coronal plane aligned with posterior nasal spine (PNS) perpendicular and axial plane passing through the palatal plane (ANS-PNS). While in the coronal plane, each CBCT image was oriented with mid-sagittal plane aligned with Nasion perpendicular. Once orientation was carried out, CBCT images and orthogonal planes were not rotated, images were only translated or scrolled, to visualize the HMS or TMS to ensure a reproducible reference plane. Then on the coronal plane, both head of miniscrew (HMS) and tail of miniscrew (TMS) were demarcated with 0.025-mm-diameter landmark. HMS was localized on the lateral superior most point, while TMS was localized on its medial superior most point. Once the landmarks are fixed on the coronal slice, it was possible to visualize them in both the sagittal and axial slices. (Fig. 1)

Transverse displacement of miniscrew was measured from HMS and TMS to the mid-sagittal plane on the coronal slice. While both vertical and sagittal displacement of miniscrew were measured on the sagittal slice as the distance from HMS and TMS to the axial and coronal planes respectively [16]. (Fig. 1) Miniscrew displacement was recorded as the difference in measurement between T0 and T1. Statistical analysis was carried out to compare the miniscrew displacement between the LLL side and control side.

### Statistical analysis

To investigate intra-rater repeatability, the same examiner (D.E) examined all parameters again, one week apart. A second examiner (E.M.) remeasured five randomly selected cases to assess inter-rater reliability. This was determined using summary statistics for the discrepancies between repeated measurements and intraclass correlation coefficients (ICCs). Normality was assessed using descriptive statistics, Q-Q plots, histograms, and normality tests. All variables had a normal distribution, therefore means and standard deviations (SD) were calculated, and parametric tests were used. Comparisons were performed using paired samples t-test with calculation of mean differences and 95% confidence intervals (CI)s. The significance level was set at *p*-value < 0.05. Data were analyzed using IBM SPSS for Windows (Version 26).

## Results

**Fig. 2:**
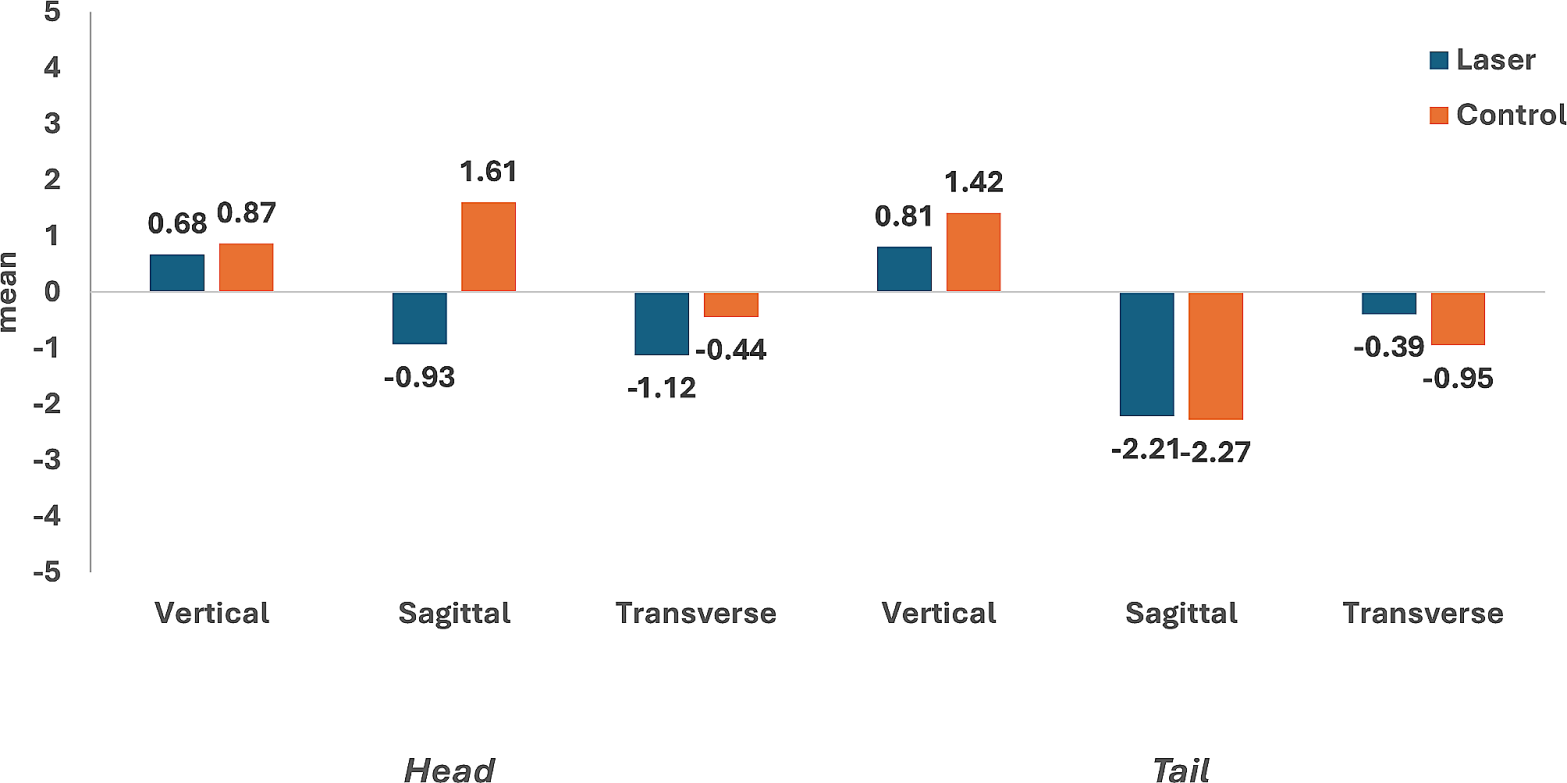
Difference in screw displacement in the laser and control sides

The ICC for intra-rater reliability indicated high reproducibility for linear measurements (0.95–0.99), in addition to excellent inter-rater reliability (above 0.9) for all measurements [25]. For both laser and control sides: mean and standard deviation for (T0) and (T1), three-dimensional position for HMS, as well as the differences between them (T1–T0) are presented in (Table 1). Moreover, same variables were recorded for TMS in (Table 2). HMS and TMS showed significant displacement in all three dimensions after four months of canine retraction regarding both sides; laser and control. An insignificant difference was detected between the mean displacement (T1-T0) of HMS and TMS between laser and control sides in the vertical, sagittal and transverse planes as shown in (Table 3) and (Fig. 2). Furthermore, no significant difference was found when comparing the mean displacement of the head and tail of miniscrews on the same side whether in experimental or control group (Table 3).

**Table 1:** Miniscrew displacement in the laser and control sides at the miniscrew head

			T0 (mm)	T1 (mm)	Difference T1-T0 (mm)	95% CI	*P* value 1
			Mean (SD)				
Head	Laser	Vertical	15.82 (4.37)	16.50 (4.44)	0.68 (0.80)	0.18, 1.19	0.01*
		Sagittal	30.70 (4.47)	32.99 (3.11)	2.29 (2.85)	0.48, 4.10	0.02*
		Transverse	31.65 (1.90)	30.53 (2.29)	-1.12 (0.67)	-1.54, -0.69	< 0.001*
		Average	26.06 (2.01)	26.68 (1.74)	0.62 (0.96)	0.006, 1.23	0.04*
	Control	Vertical	14.93 (3.33)	15.80 (3.50)	0.87 (0.99)	0.24, 1.50	0.01*
		Sagittal	30.16 (4.39)	31.77 (1.43)	1.61 (3.52)	0.63, 3.84	0.01*
		Transverse	30.75 (0.55)	30.31 (0.22)	-0.44 (0.20)	-1.74, 0.08	0.04*
		Average	25.08 (2.55)	26.44 (1.26)	1.36 (2.26)	0.07, 2.80	0.04*

T0: Immediately after miniscrew insertion, T1: Four months after canine retraction, SD: Standard Deviation, CI: Confidence Interval, *P* value 1: Comparisons between initial and final readings within each group. *statistically significant at *p* value < 0.05

**Table 2:** Miniscrew displacement in the laser and control sides at the miniscrew tail

			T0 (mm)	T1 (mm)	Difference T1-T0 (mm)	95% CI	*P* value 1
			Mean (SD)				
Tail	Laser	Vertical	10.75 (4.62)	11.56 (4.72)	0.81 (0.87)	0.25, 1.36	0.008*
		Sagittal	29.17 (1.68)	31.33 (2.06)	2.16 (0.15)	1.43, 2.09	< 0.001*
		Transverse	22.65 (2.38)	22.26 (2.62)	-0.39 (1.01)	-1.03, -0.25	0.02*
		Average	20.86 (2.31)	21.71 (2.44)	0.85 (0.61)	0.47, 1.24	< 0.001*
	Control	Vertical	11.15 (3.48)	12.57 (1.89)	1.42 (4.39)	1.37, 4.21	0.02*
		Sagittal	29.67 (1.66)	32.83 (0.61)	3.12 (1.60)	2.15, 4.18	< 0.001*
		Transverse	22.51 (2.34)	21.56 (2.62)	-0.95 (1.31)	-1.79, -0.12	0.03*
		Average	21.11 (1.76)	22.32 (0.84)	1.21 (2.08)	0.11, 2.53	0.04*

T0: Immediately after miniscrew insertion, T1: Four months after canine retraction, SD: Standard Deviation, CI: Confidence Interval, *P* value 1: Comparisons between initial and final readings within each group. *statistically significant at *p* value < 0.05

**Table 3:** Comparison of the difference in miniscrew displacement between the laser and control sides at the miniscrew head and tail

		Laser	Control	Difference	T1-T0 (mm)	*P* value 1
		Mean difference T1-T0 (SD) (mm)				
Head	Vertical	0.68 (0.80)	0.87 (0.99)	-0.18 (1.25)	-0.98, 0.61	0.48
	Sagittal	2.29 (2.85)	1.61 (3.52)	0.68 (3.02)	-1.23, 2.60	0.24
	Transverse	-1.12 (0.67)	-0.44 (0.20)	-0.68 (1.71)	-1.75, 0.41	0.17
	Average	0.62 (0.96)	1.36 (2.26)	-0.74 (1.86)	-1.92, 0.44	0.24
Tail	Vertical	0.81 (0.87)	1.42 (4.39)	-0.61 (4.50)	-3.47, 2.25	0.48
	Sagittal	2.16 (1.15)	3.17 (1.60)	-1.01 (1.56)	-2.00, 0.01	0.10
	Transverse	-0.39 (1.01)	-0.95 (1.31)	0.56 (1.28)	-0.25, 1.37	0.12
	Average	0.85 (0.61)	1.21 (2.08)	-0.35 (2.17)	-1.73, 1.03	0.46
P value 2 (head vs. tail)	Vertical	0.94	0.26			
	Sagittal	0.58	0.53			
	Transverse	0.10	0.58			
	Average	0.41	0.58			

Wilcoxon signed ranks test was used

*P* value 1: comparisons between laser and control sides

*P* value 2: comparisons between head and tail within each side

## Discussion

One major aspect influencing the outcome of orthodontic therapy is the control of anchorage or resistance to undesired tooth movement [26]. Although both intra-oral and extra-oral appliances have been employed to meet the anchorage needs, new techniques, such as implants, have been developed with the goal of obtaining adequate anchorage without the disadvantage of conventional procedures due to side effects and compliance concerns [27].

CBCT has established itself as a reliable oral and maxillofacial diagnostic imaging method, especially for orthodontic planning [28, 29]. This is mainly due to the lower radiation exposure and shorter scan acquisition time needed in comparison to conventional computed tomography (CT) scans in order to produce a satisfactory image [30]. The reference planes and landmarks can be reproduced identically owing to the simultaneous 3D reconstruction orientation and landmark demarcation on CBCTs at different time points [31]. As a result, the measurement accuracy is improved, and errors are decreased.

The results of this study showed significant displacement for both the heads and tails of the miniscrews at T1 compared to T0, regarding laser and control sides. This finding comes in accordance with previous research findings in the literature. Significant miniscrew displacement was observed by Alves et al. [16] for 41 miniscrews utilized for 200 g-loaded molar intrusion during a 5-month period. El-Beialy et al. [11] reported the displacement of 40 miniscrews that were used to retract the upper and lower canines for six months with 150 and 250 g of force. Similar results were reported with miniscrews displacement in the direction of force (mesially) and vertically (extruded) [11]. According to Pongsamart et al. [32], applying a force as low as 50 gm to the miniscrews for 3 months would result in its displacement.

However, Osman et al. [33] showed no significant displacement of miniscrews head and tail using LLL during six months of treatment. As for the control side, a significant difference was found regarding HMS while TMS did not show significant displacement. This disagreement with our results could be due to the difference in the measurement method used, as in the previous study the authors used a fixed skeletal point PNS (posterior nasal spine) on the coronal view of CBCT to measure linear distances to HMS and TMS [33]. While in the current study miniscrew displacement was assessed in all 3 planes of space (vertical, sagittal and transverse). The results showed that miniscrew’s head and tail moved downward vertically, mesial sagittally and medial transversely. This could be explained by understanding the components of the resultant force applied during canine retraction on the miniscrew, which is downward, mesial and medial [34].

The assessment of miniscrew displacement in 2 dimensions as measured from a fixed skeletal point ANS [32, 35] or PNS [33] shows movement of the miniscrew in relation to a single point, which proves to be the resultant vector of displacement; i.e. ANS or PNS. While in the current study, 3D displacement of the miniscrew was clearly illustrated through its assessment in the three planes (sagittal, transverse, and vertical).

The study’s findings showed no significant difference between the miniscrew displacement under orthodontic loads on both the control and experimental sides. This agrees with the study of Maranon Vasquez et al. [36] in which LLL produce a significant difference in miniscrew displacement between the control and study groups given the magnitude of the retraction force (150 g) and bearing time (3 months). However, an animal study by Uysal et al. [37], studied the effect of light emitting diode photo biomodulation therapy on the stability of immediately loaded miniscrews at day 1 of insertion and 21 days later, found that the irradiated group exhibited stronger stability than the nonirradiated group. This could be explained by the variations in human and rabbit skeletal structures.

Moreover, the current study demonstrated no significant difference between the displacement of the head and tail of the miniscrews in neither the laser nor the control sides, indicating parallel movement of the miniscrew. In contrast to the results of Osman et al. [33] concerning the control side which reportedly showed a significantly higher displacement of HMS compared to TMS.

### Limitations

The current study limitation is being a retrospective study testing the effect of only one laser protocol on miniscrew displacement.

## Conclusion

In this study the LLL application did not affect the displacement of miniscrews three-dimensionally. Miniscrew displacement was significant in both groups.

- LLL application in the used protocol did not affect the amount of miniscrew displacement in any of the three planes of space.
- HMS and TMS were significantly displaced in the vertical, sagittal, and transverse planes on the LLL and control sides.
- Miniscrews displacement seems to be parallel as evidenced by no discernible difference between the HMS and TMS displacements on either side.

## Data Availability

The datasets used and/or analyzed during the current study are available from the corresponding author on reasonable request.
